# Functional definitions of parietal areas in human and non-human primates

**DOI:** 10.1098/rspb.2016.0118

**Published:** 2016-04-13

**Authors:** Guy A. Orban

**Affiliations:** Department of Neuroscience, University of Parma, Parma, Italy

**Keywords:** fMRI, homology, action planning, action observation

## Abstract

Establishing homologies between cortical areas in animal models and humans lies at the heart of translational neuroscience, as it demonstrates how knowledge obtained from these models can be applied to the human brain. Here, we review progress in using parallel functional imaging to ascertain homologies between parietal areas of human and non-human primates, species sharing similar behavioural repertoires. The human homologues of several areas along monkey IPS involved in action planning and observation, such as AIP, LIP and CIP, as well as those of opercular areas (SII complex), have been defined. In addition, uniquely human areas, such as the tool-use area in left anterior supramarginal gyrus, have also been identified.

## 1. Introduction

Following the work of Mountcastle [1], it became generally accepted that the posterior parietal cortex (PPC) is involved in sensorimotor transformations underlying the planning of human actions [2]. The PPC has also been implicated in more cognitive functions, such as attention [3], working and long-term memory [4,5], numerical processing [6] and tool use [7]. One important initial step in characterizing PPC functions is the definition of parietal areas, those building blocks providing the foundation of the functional studies.

Although in exceptional circumstances neuronal activity in human PPC can be accessed directly [8], functional studies of PPC generally rely on imaging (which is limited in spatio-temporal resolution and maps neuronal selectivity only indirectly), using repetition suppression [9], or multivoxel pattern analysis [10]. The limitations of these methods have become apparent [11,12], underscoring the necessity of animal models. Animal models should be appropriate (i.e. share the brain functions under investigation). Non-human primates (NHPs)—who, like humans, use dexterous hands and mobile eyes to explore and interact with the environment—are the most valuable models for PPC. To be useful, however, any knowledge derived from the monkey brain must translate to human brain function. Hence, using the NHP to define human PPC regions requires that homologies between PPC regions be established and uniquely human areas identified.

**Figure 1.**
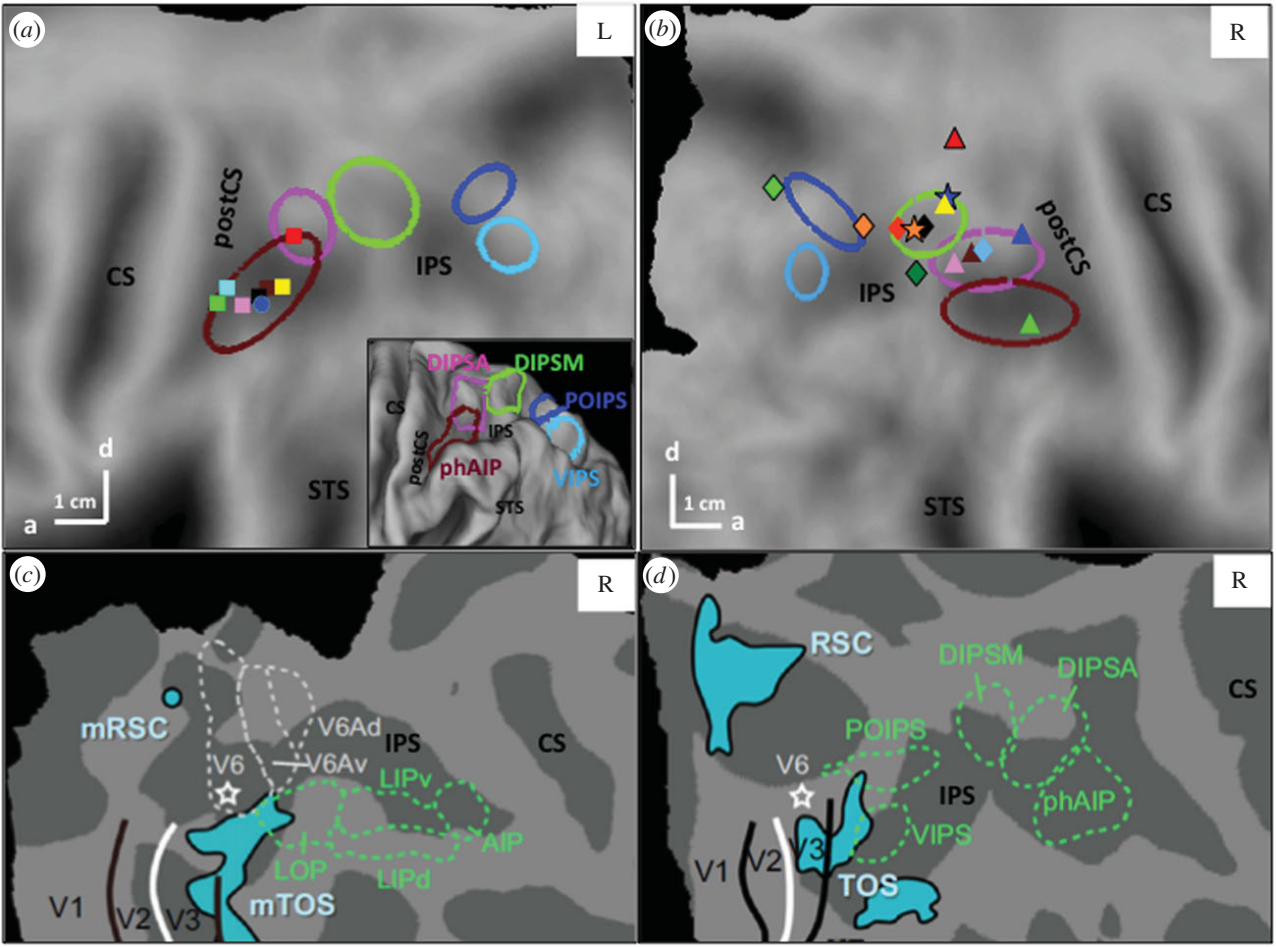
Human PPC areas hypothetically homologous to areas in the lateral bank of monkey IPS. (*a*,*b*) Confidence ellipses of five areas (brown, phAIP; pink, DIPSA; green, DIPSM; dark blue, POIPS; light blue, VIPS) in posterior parts of (*a*) left and (*b*) right flattened hemispheres (folded view in inset). (*c*,*d*) Parietal areas in (*c*) monkey and (*d*) human relative to V1–3, V6, retrosplenial cortex (RSC), transverse occipital sulcus (TOS) area and possible monkey counterparts (mRSC, mTOS); modified from Vanduffel *et al*. [18]. LOP is synonymous with CIP; IPS, intraparietal sulcus; CS, central sulcus. Symbols in (*a*)—local maxima (LM) for grasping: circle: [19]; squares: [20] (yellow), [21] (red), [22] (black), [23] (green), [24] (pink); visuo-tactile: [25] (brown); and 3D hand orientation: [26] (blue). In (*b*)—stars: LM of human LIP using spatial attention (orange, [27]) or saccades (blue, [28]); diamonds: other saccade LM: [15] (light and dark green), [29] (orange and red), [16] (black) and [28] (light blue); triangles: proposed human VIP: [30] (yellow), [31] (red), [32], (pink), [33] (green), [34], (blue), [16] (brown). Last two LM: visuo-tactile, others optic flow. Putative VIP LM stretch over 40 mm in medio-lateral direction, putative LIP LM over 40 mm in rostro-caudal direction, preventing the computation of useful confidence ellipses.

Monkey single-cell studies have established that LIP, AIP and the parietal reach region encompassing MIP and V6A are involved in planning saccades, grasping and reaching, respectively [13]. However, applying single-cell NHP results directly to human fMRI involves changing both experimental technique and species [14], and often incorrectly assumes that an area is unique in having neurons endowed with a given property. For example, activation by saccades is often considered the signature of LIP [15,16], although single-cell [17] and imaging [18] studies have shown more widespread activation of monkey PPC. Indeed, saccades activate a substantial part of human PPC (figure 1). Such difficulties can be avoided using fMRI in alert monkeys as an intermediate step between human fMRI and NHP single-cell studies. This review builds on such parallel imaging studies.

Establishing homologies between cortical areas in humans and macaque monkeys considers only a very small subset of primates, while ideally one would examine multiple species within this order. The alternative is to consider as many different properties of the areas under investigation as possible in both species [35]. In NHP, visual cortical areas are defined by four criteria: (i) cyto- and myeloarchitectonics, (ii) anatomical connections with other areas, (iii) retinotopic organization, and (iv) functional properties. We suggest using those criteria to establish cortical homologies, adding area topology—localization with respect to neighbouring areas—as a valuable fifth criterion [35], following the tradition of comparative anatomy. While progress has been made in mapping cyto- and myeloarchitectonic architecture in the human brain, methodologies differ considerably from those classically used in monkeys, thus far preventing systematic comparison. Although diffusion tensor imaging (DTI) [36] was potentially seen as a measure of connectivity between areas, recent comparative studies in the monkey question the value of DTI as a proxy for tract tracing in animals [37]. Thus, the chief criteria for establishing homologies remain assessing retinotopic organization and as many functional properties as possible, both of which can be adequately tested by parallel imaging of macaque and humans. These can be supplemented by topological arguments, and, where possible, by cyto- or myeloarchitectonic data.

Human cerebral hemispheres have 9.2 times the surface area of macaque hemispheres [38,39]. The number of human cortical areas is estimated at 150–200, a 1.3-fold increase over monkeys (130–140 areas per hemisphere), suggesting that new areas have appeared in humans to support typically human behaviour, such as tool use or language. In this review, we concentrate on two sets of parietal areas as candidates for homologous areas: those along the intraparietal sulcus (IPS) and the parietal opercular areas. Conversely, species differences are documented in the inferior parietal lobule (IPL).

## 2. Similarities along the intraparietal sulcus

We have described five regions along human IPS whose homology is becoming clear (figure 1). Because activation studies mainly yield local maxima (LM), these areas were defined [40] as confidence ellipses surrounding these maxima (figure 1*a,b*). The four caudal ellipses representing motion-responsive regions [41] follow the dorsal/posterior bank of the IPS: the dorsal IPS anterior (DIPSA), dorsal IPS medial (DIPSM), parieto-occipital IPS (POIPS) and ventral IPS (VIPS), rostrally to caudally. Rostral to DIPSA, we described the putative homologue of AIP (phAIP) from maxima of motor activation during grasping and multimodal activations (see legend, figure 1). These latter LM cluster very well, allowing computation of a confidence ellipse (figure 1*a*), unlike activations by saccades identifying LIP, or activations by visuo-tactile convergence or optic flow, identifying VIP (figure 1*b*). We propose (table 1) that phAIP and DIPSA correspond to anterior (motor), and posterior (visual) parts of monkey AIP, respectively [18,42], while DIPSM corresponds to anterior LIP and VIPS to monkey CIP (figure 1). These areas are discussed together, stressing topological relationships. Note that AIP–CIP are located along the lateral/ventral bank of monkey IPS, DIPSA-VIPS along the dorsal/medial bank of human IPS, in agreement with Grefkes & Fink [43]. In both species, these regions lie rostral to the V6 and V3A complexes. The homology of POIPS is less clear: it probably corresponds to an area on the medial bank of monkey IPS rostral to V6/V6A.

**Table 1.** Proposed homologies of IPS regions.

monkey area	human area(s)	criteria
AIP	phAIP+vDIPSA	6/8 functional tests of table 2
anterior LIP	DIPSM	8/9 functional tests of table 2
VIP	dDIPSA	visuo-tactile sensitivity optic flow sensitivity intrusion-into-peripersonal-space sensitivity numerosity selectivity size selectivity topological relationships
CIP	VIPS (V7/V7A)	retinotopy 3D shape-from-disparity sensitivity 2D shape sensitivity topological relationships

**Figure 2.**
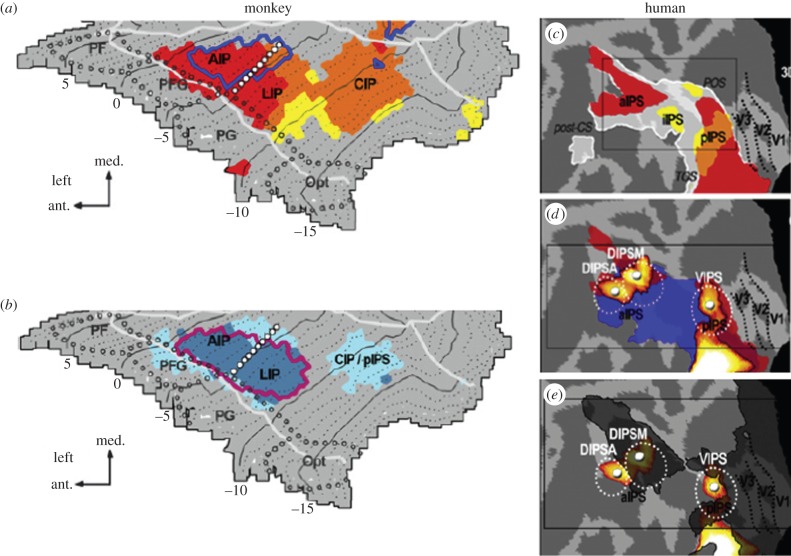
Parallel imaging of 2D and 3D shape sensitivity: (*a*,*b*) lateral bank of left monkey IPS (from [45]) (*a*) 3D shape-from-disparity, (*b*) 2D shape sensitivity; (*c*–*e*) left human IPS (from [46])—(*c*) 3D shape-from-disparity; (*d*) 2D shape sensitivity; (*e*) saccade sensitivity (dark hatching); colours: see text. In (*a*,*b*) white dotted lines indicate the AIP/LIP borders derived from the saccade-related activation.

As stated, establishing a homology necessitates examining as many functional characteristics as possible. Indeed, the property initially considered, sensitivity to three-dimensional (3D) structure from motion (SFM) using random line stimuli, revealed marked differences between human and monkey PPC [44]. In many other respects, however, the lateral bank of monkey IPS and dorsal bank of human IPS are functionally similar [45,46]. Rostrally to caudally, three regions emerge along the banks of IPS in both species: a rostral 3D shape-from-disparity (SFD)-sensitive region (red in figure 2*a*,*c*), a mid-region with sensitivity to disparity but not 3D SFD (yellow), and a caudal region with mixed sensitivities for 3D SFD and simple disparity (orange/red). The rostral region corresponded to a single, large two-dimensional (2D) shape-sensitive region encompassing posterior AIP and anterior LIP, and to two 2D shape-sensitive regions, DIPSA and DIPSM in humans. The caudal region, also 2D shape-sensitive, corresponded to CIP in monkeys and VIPS in humans (figure 2*b*,*d*). In both species, saccade sensitivities differentiated two components in the rostral region: anterior LIP/posterior AIP in the monkey and DIPSA/DIPSM in humans (figure 2*b*,*e*). These and similar studies [42] indicate that DIPSM and anterior LIP share 8 of 9 characteristics investigated, 3D SFM being the exception (table 2). Similarly, posterior AIP and DIPSA share 6 of 8 characteristics. Significantly, combined single-cell and fMRI experiments in monkey [47] suggest that 3D SFD activations in DIPSA and DIPSM actually correspond to two neuronal clusters selective for 3D SFD in the rostral lateral bank of monkey IPS. This underscores how parallel imaging in humans and NHP disclose neuronal operations in the human brain.

**Table 2.** Criteria for homology of DIPSM and anterior LIP.

criterion	anterior LIP	DIPSM
central representation^a^	+	+
motion sensitivity^b^	+	+
2D shape sensitivity	+	+
3D shape from disparity (random lines) sensitivity	+	+
3D shape from disparity (surfaces) sensitivity	+	+
3D shape from motion sensitivity	−	+
saccade sensitivity^c^	+	+
observed-action sensitivity	+	+
topological sequence for 2D shape and disparity	+	+

^a^Not tested in AIP.

^b^Fails in AIP.

^c^Absent in both AIP and DIPSA.

**Figure 3.**
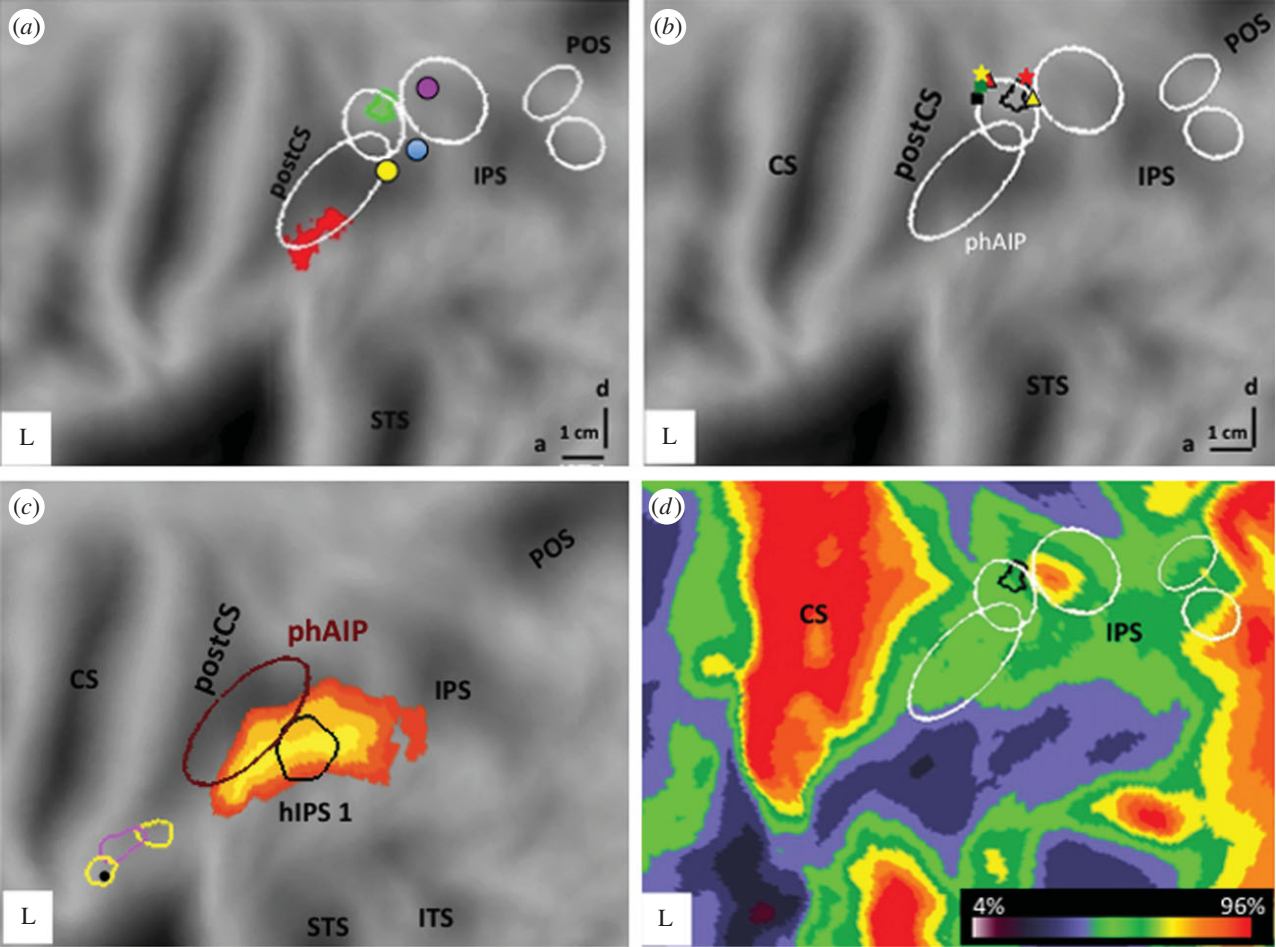
Anterior IPS regions on left flatmap: (*a*) voxels specific for observation of manipulative (red, phAIP) and interpersonal actions (green, dDIPSA); (*b*,*d*) dDIPSA (black outline) relative to local maxima of studies as indicated (*b*), and to myelin density peak near middle IPS (*d*). (*a*,*b*,*d*) Modified from Ferri *et al*. [54]. Dots in (*a*)—IPS3 (purple), 4 (light blue) and 5 (yellow) from Konen *et al*. [55]. Symbols in (*b*)—stars: centre of numerosity (yellow) and size (red) maps averaged over four subjects from [56,57]; other symbols: LM of [34] (red), [32] (yellow), [58] (black), [59] (green). (*c*) Parietal part of human-specific resting-state network (red to yellow voxels) compared with hIPS (black outline): from [60], and outline of left anterior SMG region specific for human tool use: from [61] (pink), [62] (yellow) compared with phAIP (brown). Black dot indicates LM of interaction execution/tool [63].

Initially, only phAIP was considered homologous to monkey AIP, but this was based upon motor response during grasping and somato-sensory convergence, characteristics of rostral AIP [48,49]. This supports phAIP *plus* DIPSA being the human counterpart of monkey AIP [46]. The devotion of such a large region to planning hand actions is consistent with their importance in the human motor repertoire. The homology of phAIP/DIPSA with monkey AIP is further supported by action-observation studies in both species. Nelissen *et al*. [50] showed AIP activation in monkeys observing grasping actions, in agreement with Pani *et al*. [51] and Maeda *et al*. [52]. Similarly, three human studies [40,53,54] documented phAIP sensitivity to observed manipulative actions (figure 3*a*).

The phAIP, DIPSA and DIPSM regions are mere confidence ellipses whose sizes depend on the variability of the LM locations defining them. While subsequent work suggests that phAIP is indeed functionally homogeneous, this may not be true of DIPSA. A recent study [54] suggested that dorsal DIPSA (dDIPSA) is functionally distinct from the ventral part located in the extension of phAIP (figure 1). Because the activation by observed interpersonal actions (figure 3*a*,*b*) was located between other activations reflecting typical VIP characteristics such as somato-visual convergence [34,59], intrusion into peripersonal space [58] and optic flow [32], we have proposed that dDIPSA may correspond to VIP, while only ventral DIPSA (vDIPSA) corresponds to posterior AIP. The activation by observing interpersonal actions was thus interpreted to reflect the intrusion or movement of the target person in the peripersonal space of the actor. Indeed, visuo-tactile VIP neurons also react to visual stimuli in the peripersonal space of the experimenter [64]. This identification is also supported by the overlap with size and numerosity maps [56,57], as VIP neurons are selective for numerosity and size [65,66]. It is also consistent with the proximity of dDIPSA to the myelin density peak in dorsal IPS (figure 3*c*) [67], which may correspond to ventral LIP [68]. Our most recent work suggests that DIPSM may also require splitting into a ventral part and a dorsal part extending the homologue of VIP more caudally. Such subdivisions are unsurprising, because areas in lateral bank and fundus of monkey IPS occupy narrow, parallel strips [68].

**Figure 4.**
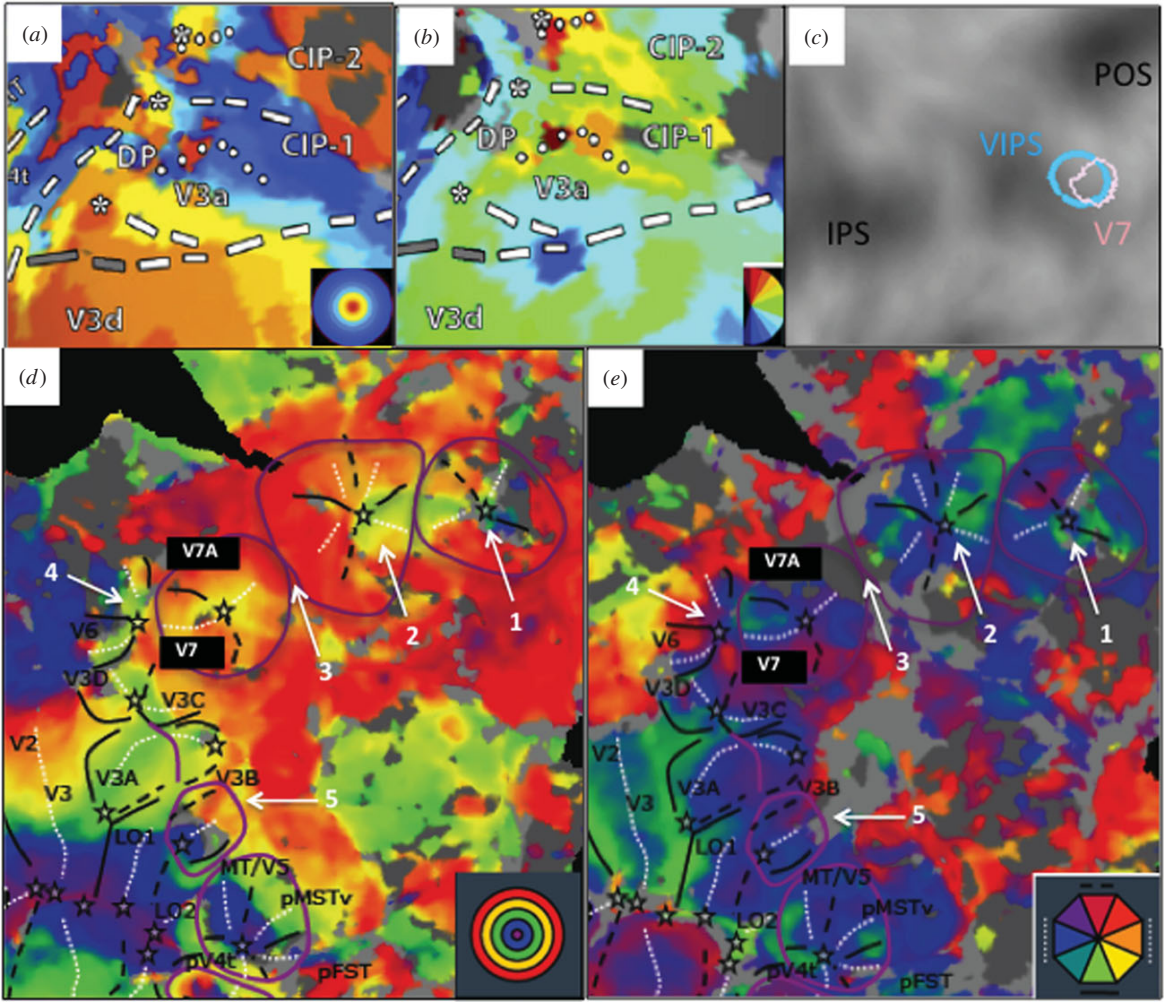
Retinotopic organizations: human V7/V7A cluster homologue of monkey CIP. (*a*,*b*) Eccentricity and azimuth CIP maps in the monkey (from [69])—stars: central representation, dotted lines: horizontal meridians, dashed lines: vertical meridians. (*c*) Overlap between functional VIPS and retinotopic V7: the rostral part of VIPS probably corresponds to V7A; (*d*,*e*) eccentricity and azimuth maps of posterior right hemisphere of subject 1 (same as in fig. 1 of [67]) centred on the V7/V7A cluster. Purple lines: far eccentricity borders of clusters; black stars: central representation, full and stippled black lines: lower and upper vertical meridians, white stippled lines: horizontal meridians. Arrows indicate rostral C clusters (1, 2), eccentricity ridge rostral to the V7/V7A cluster (3), centre of V6 (4) and a retinotopic map potentially corresponding to monkey DP (5).

Progress establishing homology between monkey CIP and human VIPS has been slower. Support, beyond that reviewed above, comes chiefly from retinotopic studies. A region in posterior PPC, dorsal to human V3A/B, overlapping posterior VIPS (figure 4*c*), has long been designated V7 [70] or IPS0 [55]. A recent study [71] using the stereoscopic stimuli very effective in caudal human IPS [72] has revealed a central (C) cluster (two areas sharing a central representation) in the occipital part of IPS, rostral to V6 and separated from rostral clusters by a broad representation of far eccentricities (figure 4*d*,*e*). We propose that this V7/V7A cluster is the retinotopic counterpart of functionally defined VIPS. Its organizational features strongly resemble those of monkey CIP, which also corresponds to a C cluster of two areas (figure 4*a*,*b*) [69] and is separated from anterior LIP by a representation of the far periphery in posterior LIP. Thus, there is some support for identifying VIPS with monkey CIP, both of which comprise two areas.

## 3. Similarities in the parietal operculum

**Figure 5.**
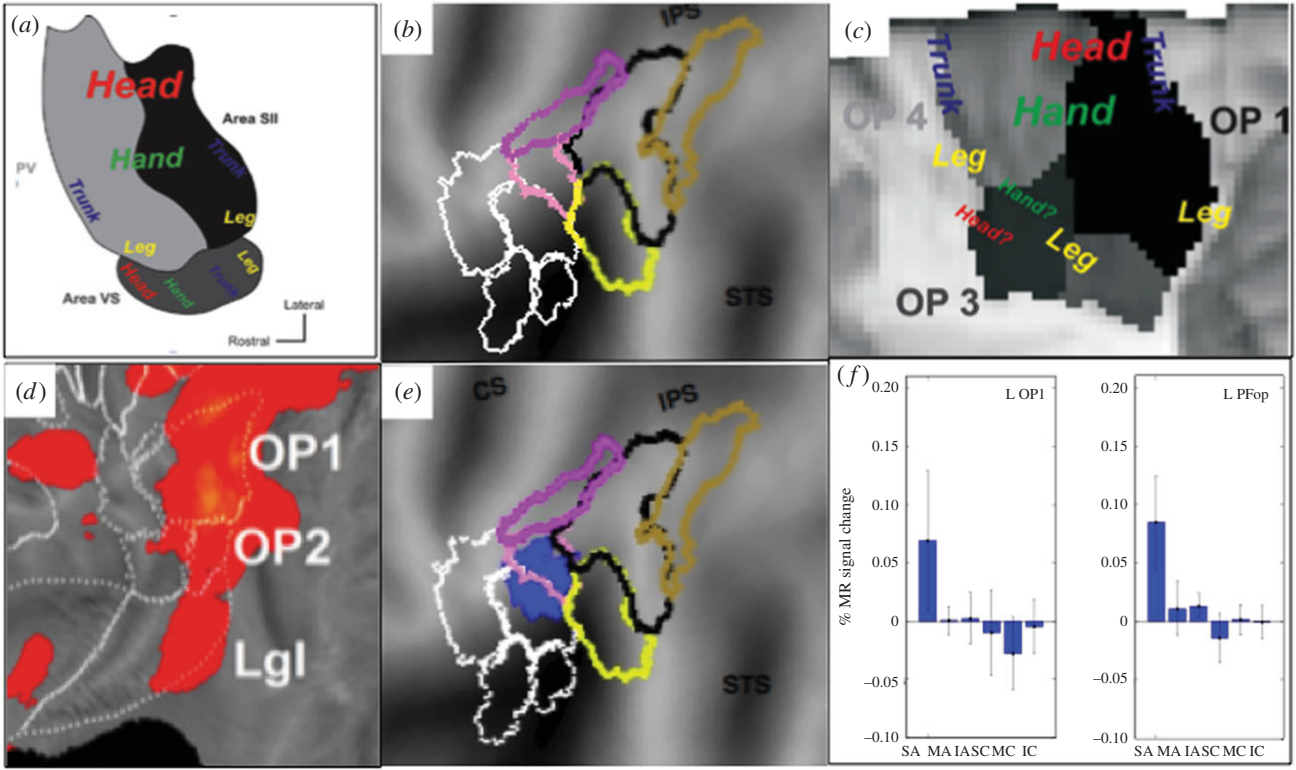
Homologies of parietal opercular areas. (*a*) Somatotopy of monkey areas (from [75]); (*b*) flatmap of left operculum indicating the four opercular areas (white outlines) and the five PF areas (coloured outlines); (*c*) somatopic organization of OP1, OP3 and OP4 [75]; (*d*) response to electrical stimulation median nerve in OP areas (from [76]); (*e*,*f*) activation sites for observation of skin-displacing action (*e*) and their activity profiles (*f*) from Ferri *et al*. [54].

Although strictly speaking the parietal operculum is not part of PPC, which includes only areas 5 and 7 in the monkey and 5, 7, 40 and 39 in humans, we discuss opercular homology here because it also contains higher-order sensorimotor areas. Eickhoff *et al*. [73] described four cytoarchitectonic regions in the operculum, labelled OP1-4, located anterior to the various PF regions [74] constituting the rostral IPL (figure 5*a*). Using somatotopic mapping with fMRI, Eickhoff *et al.* [75] provided evidence that OP1 and OP4 correspond to monkey S2 and PV (figure 5*b*,*c*). They suggested that OP3 corresponds to the third somatotopic map in the monkey area VS and speculated that remaining OP2 might be vestibular in nature. Recently, we have begun using stereo EEG, intracerebral recordings of local field potentials in epileptic patients, to complement our fMRI studies. Recording from many patients, reconstructing lead locations and warping hemispheres to a template has allowed us to reconstruct four-dimensional maps of human cortex, combining millimetre spatial localization with millisecond time resolution, in response to median nerve stimulation [76]. This study has shown that OP2 processes somato-sensory information as much as OP1 or OP4. Hence further work is needed to understand the homology of area VS, as some monkeys have two VS areas [77], but the evidence relating OP1 and OP4 to S2 and PV is rather convincing.

Recently, we [54] have shown that OP1, and neighbouring PFop, are activated by the observation of skin-displacing actions, such as rubbing or scratching (figure 5*d*). Control tests indicated that this activation reflects the dynamic nature of the actions observed, not the viewing of tactile contact. The co-activation of OP1 and PFop (figure 5*e*) was reminiscent of the robust projections between monkey S2 and PF [78], whereby PF provides output to OP1 (human S2), complementing OP4 (human PV) in this role. In fact, we have suggested [54] that OP1 and PFop contributions to the observed-action activation may correspond to the sensory and motor parts, respectively, of the transformation underlying planning of skin-displacing action, resembling the respective roles of posterior and anterior AIP.

## 4. Species differences in the inferior parietal lobule

Warping monkey cortex to its human counterpart, then performing cluster analysis of the resting-state networks of the two species, Mantini *et al*. [60] found three human networks with no functional or topological monkey counterparts. Two of these evolutionarily novel networks were lateralized, but both included a common IPL region (figure 3*d*). This novel IPL region overlapped with hIPS, implicated in numerical processing [6] and with anatomical regions undergoing intense evolutionary expansion in humans [79]. Although the hIPS region has been associated with monkey VIP [80], recent data indicate that numerosity and size maps [56,57], consisting of voxels tuned to small numerosities or size, like VIP neurons [65], are located dorsal to hIPS, overlapping the proposed homologue of VIP (figure 3*b*). Hence, I suggest that human PPC hosts two numerosity processing regions separated by phAIP/vDIPSA: one common with the monkey in dDIPSA, supporting subitizing, and another specifically human, in hIPS, supporting counting.

Another functionally defined region exemplifying cortical expansion in human IPL is the left anterior supramarginal (aSMG) tool-use region (figure 3*d*) [61,62]. This region responds specifically to observation of tool actions, but not hand actions with similar goals, unlike phAIP, which responds to either. Videos used to define aSMG yielded no such specific IPL activation in monkeys, even after extensive training using pliers or rake, the tools featuring in those videos [61]. This aSMG region corresponds precisely to a region active when humans use tools [63]. We have suggested that this region, corresponding to cytoarchitectonic PFt [74], is a typically human area, underlying the development of tool use in humans [81]. Most likely, the use and creation of tools, technology, is based on the interaction of this area with several others in PPC and temporal lobe [82].

It is unlikely, despite its expansion, that all human IPL is evolutionary novel. For example, it has been recently shown that a region in monkey PG connected to the hippocampus is activated by the retrieval of the first of several previously seen items [5], very much like the human angular gyrus [83].

## 5. Discussion and conclusion

The studies reviewed here have begun to illuminate challenging questions concerning homologies of macaque and human parietal regions, and many objectives defined a decade ago [35] have now been met. Critical elements were parallel imaging in these two species and employing multiple functional criteria, revealing a substantial number of homologous PPC areas. This approach resolves the translational question of how knowledge accumulated through invasive experiments in macaques can be applied to humans, where investigations are more limited for ethical reasons. Monkey single-cell studies can thus provide particularly valuable information about neuronal mechanisms underlying human behavioural competences. For example, the homology between phAIP/vDIPSA and monkey AIP implies that the canonical and mirror neurons observed in single-cell studies [49,52] also exist in this human area.

The studies also suggest two avenues for further progress. One is to leverage the topological relationships between areas, which are generally retained across species. A set of homologous regions, once identified, can provide a seed for extending functional correspondences, and ultimately homology, to neighbouring regions. For example, regions dorsal to DIPSM are involved in the execution and observation of reaching [84], suggesting homology with macaque MIP and V6A in the medial bank of IPS, befitting topological relationships in both species. Second, some studies reviewed here suggest action observation can serve as proxy for action planning and execution. This may circumvent the limitations on the range of sensorimotor transformations observable in a monkey sitting in a chair with its head fixed, or in human subjects lying supine in a scanner (largely grasping, reaching and saccades). Moreover, videos are easily shown to both monkeys and humans, facilitating attribution of sensorimotor transformations to discrete PPC regions and establishing homologies.

Finally, taking a broader perspective, the few PPC regions present in rodents [85] are probably involved in locomotion and coarse use of the forepaws. These areas probably correspond to the medial wall of primate PPC, though they surely have undergone substantial modification to accommodate the navigational needs [86] of primates, especially bipedal humans. NHP Brodmann areas 5 and 7 have been added to those ancestral PPC regions in a medial-to-lateral direction for the sophisticated control of mobile eyes and dexterous hands. This medio-lateral trend was further amplified in humans with the expansion of IPL, generating areas 39 and 40, to control vocal and other communication as well as the use of artefacts, extending the potential of biological effectors.
